# Modeling of Polycrystalline Material Microstructure with 3D Grain Boundary Based on Laguerre–Voronoi Tessellation

**DOI:** 10.3390/ma15061996

**Published:** 2022-03-08

**Authors:** Xingshuai Zheng, Tengfei Sun, Jixing Zhou, Rupeng Zhang, Pingmei Ming

**Affiliations:** School of Mechanical and Power Engineering, Henan Polytechnic University, Jiaozuo 454000, China; 13103693199@163.com (T.S.); 15538918806@163.com (J.Z.); zrp9126@163.com (R.Z.); mingpingmei@163.com (P.M.)

**Keywords:** polycrystalline material, microstructure, Laguerre–Voronoi tessellation, grain boundary

## Abstract

Voronoi tessellations are shown to be statistically representative of polycrystalline microstructures, which have been widely accepted for the modeling of microstructures of metallurgic and ceramic materials. In this paper, a new implementation of the Voronoi diagram in Laguerre geometry is presented for the generation of numerical models of polycrystalline microstructures, where the size and shape of the grains can be controlled, and the 3D grain boundaries can be modeled with a specified thickness. The distribution of grain sizes in the models is fitted to a lognormal distribution, compared with the normal distribution in the Voronoi tessellation methods. Finally, statistical analyses of grain face and grain size distribution are performed with the models, and the macroscopic elastic properties of polycrystalline ceramic materials are simulated to verify the capability of the presented method.

## 1. Introduction

Polycrystalline materials are widely used as an important material in many fields such as construction, medical, and aerospace. Recent studies have demonstrated that the deformation, damage, and even fracture of polycrystalline materials at the macroscopic level depend on the microstructural characteristics of the materials, so it is important to understand the deformation mechanism of polycrystalline materials at the grain level to study the mechanical properties of these materials in the macroscopic field. The microscopic characteristics of polycrystalline materials include the single crystal behavior, the distribution of grain sizes and grain orientations, and the polycrystal morphology, etc. Therefore, the development of optimization techniques for polycrystalline material design requires a topological structure model with appropriate description of the statistical, topological, and physical characteristics of the microstructure.

Modeling techniques for polycrystalline material microstructures have developed rapidly in recent decades, either by material testing methods based on one-to-one experimental measurements, or by computer simulation methods based on statistical laws and algorithms.

With the development of materials testing technology, experimental measurement techniques have been used to measure real microstructural topological information of materials (e.g., grain size distribution, first neighborhood number, and grain morphology). Ultrasonic excitation-based nondestructive detection techniques that rely on the reflections of high frequency acoustic waves are not very effective for imaging grains in metals. Subsequently, the application of X-ray computed tomography technology solved this problem. Simonovski et al. established a microstructure model of stainless steel based on X-ray diffraction contrast tomography data [1]. This method achieved the visualization of polycrystalline material microscopic modeling. However, complemented techniques (e.g., electron backscatter diffraction (EBSD)) must be used to identify the distribution of grain orientation.

Introduced more recently, the 3D real tissue modeling method based on serial sectioning techniques is one of the most prospective methods due to its high-fidelity structure of the materials. This technique builds 3D microstructure models in 3D space by stacking the two-dimensional microstructure information obtained following serial sectioning. This technique still needs an EBSD system to obtain the grain orientation [2]. Generally speaking, all these modeling methods based on experiments require high research funding and must deal with the huge amounts of data generated during the experiments, which require complex post-processing techniques. Furthermore, the transformation process between 2D and 3D models generates non-unique topological relationships due to the overlap of grains.

Due to the aforementioned difficulties in experimental methods and the difficulty in generating large-scale grains, computer modeling and simulation techniques have gained researchers’ attention with their advantages in investigating the microstructure of polycrystalline materials. Furthermore, they bring great convenience to the establishment of finite element models in the subsequent simulation of deformation and damage of crystalline materials.

Initially, researchers investigated the micro-mechanics of polycrystalline materials with numerical simulations based on regular two-dimensional shapes, including Baczmanski [3], who proposed thousands of square grains to simulate the residual stresses in steel in plastic deformation, followed by Ortiz [4], who used ortho-hexagonal grains for residual stress simulations and concluded that at least two hundred grains were required to make the calculations more accurate. These regular shape grain models are simple to establish and easy to mesh. However, during the crystallization processes, because of the mutual resistance between adjoining grains, the grains spontaneously form polyhedral irregular grains. The above square or ortho-hexagonal grain models do not match with the actual grain shapes and cannot reflect the grain irregularity and the grain inhomogeneity deformation of inner materials. Among the numerical techniques developed to generate representative models of polycrystalline microstructures, Voronoi tessellation techniques are generally considered to offer an excellent compromise between representativeness and simplicity of formulation. Therefore, the modeling methods based on the Voronoi diagram have been widely used in the field of polycrystal modeling in recent years.

With the improvement of computing power, polycrystal modeling methods based on the Voronoi diagram were developed, and polycrystal models were developed from simple 2D polygons to 3D polyhedral models that are more consistent with the actual grain morphology. One of the most representative 3D polycrystal modeling methods was proposed by Quey et al., which is a modeling method for generating 3D random models of large-scale grain polycrystals [5]. This method uses the open-source software Neper to generate the topological information of the grain model and reconstruct the polycrystal model by topological information in reverse topology in CAE software. Neper runs on any Unix-like system, which requires a large number of commands to configure the system and compile the software, and the process is troublesome and time-consuming for the researchers.

In recent years, Liang et al. generated the basic Voronoi cells information with MATLAB, stored the cells’ information as data files according to a certain order, and imported the data files into ABAQUS through the Python interface to establish polycrystalline microstructure models [6]. This method implements the Quey.R method in Windows, reducing the researchers’ usage requirements. A large amount of grain information data is generated through the Voronoi diagram function in MATLAB, and then the polycrystalline microstructure can be modeled by reverse topological reconstruction using grain information (such as vertices, lines, surfaces, bodies) in the ABAQUS package. It remains an arduous task to establish polycrystalline models.

There are several variations of the Voronoi tessellation algorithm available in the open literature; Poisson–Voronoi, Hardcore–Voronoi, and Laguerre–Voronoi formulations are widely used to statistically represent the real polycrystalline microstructures. Compared with the first two variants that have only limited control over the shape and size of the cells, the Laguerre–Voronoi formulation imposes constraints on the initial state [5], thus allowing the formulation of the tessellation to be modified more deeply. The Laguerre–Voronoi formulation can be used to model a wider range of grain structures (e.g., metals [7], foams [8], and granular matter [9]).

In addition, it is universally acknowledged that grain boundaries (GBs) govern many properties of polycrystalline materials. With the decrease of grain size, the volume ratio of grain boundary structures increases with more significant effects on macroscopic material properties, especially in the nanomaterials [10,11]. However, the above-mentioned research works model grain boundaries as ideal interfaces without thickness that does not consider the 3D solid structure of grain boundaries.

In this paper, a new implementation of the Voronoi diagram in Laguerre geometry is presented for the generation of numerical models of polycrystalline microstructures. This method directly models the grains in CAD software by repeating cutting of the corresponding cell with several cutting planes, which are created by the Voronoi diagram. The 3D grain boundary structure can be constructed in the microstructure models. There is no need for cumbersome data processing and reverse topology reconstruction. Moreover, the resulting models can be directly imported into various computer aided engineering (CAE) software (e.g., ABAQUS, ANSYS, COMSOL) for the simulation and analysis of polycrystalline materials. This paper is organized as follows: In Section 2, the procedure of modeling polycrystalline microstructures is described. In Section 3, the finite element homogenization method based on the concept of representative volume element (RVE) is implemented to evaluate the effective elastic properties of the ${\mathrm{Al}}_{2}O_{3}$ ceramic material. In Section 4, statistical analyses of grain face and grain size distribution are performed with the models, and the macroscopic elastic properties of polycrystalline ceramic materials are simulated to verify the capability of the presented method.

## 2. Polycrystal Modeling

Voronoi tessellations and its variants provide an analytical formulation to reproduce the non-regularity of polycrystalline morphologies. According to Boots’s hypothesis on crystals [12],

- All crystalline nuclei have the same weight, appear at the same time, and remain fixed in the same location during the growth process.
- The growth is uniform and isotropic with a constant rate.
- Grain growth in a direction stops when two grain boundaries contact each other.
- In the aggregate there are no voids in between the grains or grain overlapping.

Among all the numerical techniques for generating representative models of polycrystalline microstructures, the Voronoi-based tessellation techniques are generally considered to offer an excellent compromise between representativeness and simplicity of formulation. Additionally, the straight edges and the planar faces of the grains are advantageous for further spatial discretization in finite element models.

### 2.1. Voronoi Tessellation

The Voronoi diagram is essentially a spatial partitioning structure. In three-dimensional space $D^{n}$, given a finite set of N points $P=\left\{p_{1},p_{2},\ldots p_{n}\right\}$ (hereinafter called nuclei), the space is divided into a series of regions using the nucleus. Each region is the set of nearest points to the nucleus, and in this way a boundary is set for the region to achieve spatial partitioning. The set is defined as follows:

(1) $$V_{i}=p\in D^{n}\left|d\left(p,p_{i}\right)<d\left(p,p_{j}\right),\right|j\neq i\;,i=1,2,\ldots n$$

where p is any point in the *n*-dimensional space $D^{n}$, and $d\left(p,p_{i}\right)$ denotes the distance from point p to $p_{i}$.

Voronoi tessellations have been applied to spatially discretize models in a variety of fields such as astronomy, materials science, and biology. It is obvious that Voronoi tessellation has obvious advantages in simulating the grain growth results of polycrystalline materials, such as metals or ceramics, and establishing the finite element model of polycrystalline materials. This method partitions the space to form a 3D structure by randomly creating a nucleus in the 3D space. The method is simple, stores less data, and has mature algorithms through recent development, which makes it easier to achieve the establishment of polycrystal microstructure models.

With the development of Voronoi tessellation in the field of polycrystalline microstructure modeling, the limitations of this algorithm are exposed: the grain sizes of polycrystalline models being normally distributed and not necessarily compatible with real polycrystalline materials, and the singularity and non-tunable nature of Voronoi tessellation in polycrystal modeling. In particular, they do not represent a large range of real grain sizes and the presence of large grains within the microstructure [13]. Therefore, it is imperative to design a core algorithm that can satisfy the requirements of polycrystal modeling.

### 2.2. Laguerre–Voronoi Tessellation

The Laguerre–Voronoi diagram is studied on the basis of the Voronoi diagram. The Laguerre–Voronoi diagram divides the 3D space into N regions by defining Laguerre distances, which are composed of the nearest points to the nucleus Laguerre distance. Laguerre distance is defined as follows:

(2) $$d_{L}\left(p,C_{i}\right)=d{\left(p,p_{i}\right)}^{2}-\gamma_{i}^{2}$$

The Laguerre–Voronoi diagram is defined by the following equation:

(3) $$V_{i}=p\in D^{n}\left|d_{L}\left(p,C_{i}\right)<d_{L}\left(p,C_{j}\right),\right|j\neq i\;,i=1,2,\ldots n$$

where $\gamma_{i}$ and $p_{i}$ are the radius and center, respectively, of any sphere $C_{i}$ in space $D^{n}$; $d_{L}\left(p,C_{i}\right)$ denotes the Laguerre distance from point p to circle $C_{i}$.

Thus, the N-dimensional space is divided into *n* Laguerre–Voronoi regions and the corresponding boundaries, which constitute the Laguerre–Voronoi diagram. Compared with the Voronoi model, the grain size of the Laguerre–Voronoi model is controlled by the Laguerre distance, which changes the distribution of grain size so that the grain size is more consistent with the real distribution of grains.

From the mathematical description of the Laguerre–Voronoi tessellation, the overlap between the power circles can be avoided by imposing a non-overlapping condition on the spheres representing the weights of the nuclei. The Random Close Packing of Spheres (RCPS) model is usually adopted as the conditioning method [14,15]. This conditioning method, adopted in this paper, generates dense three-dimensional packing of spheres with an arbitrary radii distribution.

**Figure 1:**
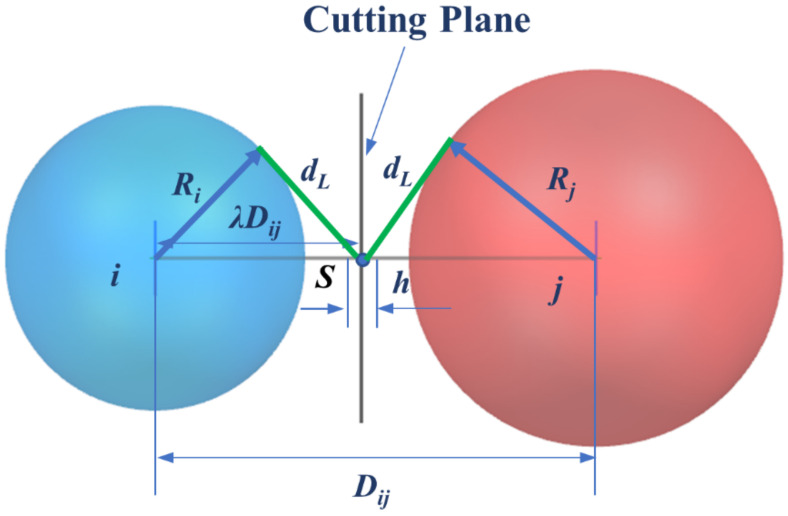
Schematic of the improved Laguerre–Voronoi tessellation, $R_{i},\;R_{j}$ are the radii of the power circles corresponding to the adjacent nucleus, $D_{ij}$ is the distance between nuclei, h is the thickness of the grain boundary, and the point S is the location of the cutting plane between two nuclei, λ is the ratio of the distance from point S to nucleus i to $D_{ij}$, $d_{L}$ is the tangential distance from point S to two spheres.

Moreover, polycrystalline microstructure models with 3D grain boundaries have also been established based on the Laguerre–Voronoi tessellation formulation. As shown in Figure 1, $R_{i},\;R_{j}$ are the radii of the power circles corresponding to the adjacent nucleus, $D_{ij}$ is the distance between nuclei, h is the thickness of the grain boundary, and the point S is the location of the cutting plane between two nuclei. According to the Laguerre–Voronoi diagram, the distance ratio from the point S to the nucleus i is denoted as λ, and the tangential distances from the point S to the two spheres are equal. Thus, the position of the cutting plane can be calculated by the derivation of the equation as follows:

(4) $$d_{L}^{2}={\left(\lambda D_{ij}\right)}^{2}-R_{i}^{2}={\left(1-\lambda \right)}^{2}D_{ij}^{2}-R_{j}^{2}$$

deduce that

(5) $$\lambda =\frac{R_{i}^{2}-R_{j}^{2}+D_{ij}^{2}}{2D_{ij}^{2}}$$

It is evident that when the grain boundary thickness is set to zero where the grain boundary is considered as ideal interfaces without thickness, the distance from the cutting plane to the nuclei is $\lambda D_{ij}$, and if the thickness of the grain boundary is set to h, this value is equal to $\lambda D_{ij}-h/2$. In addition, when the RCPS model has the same radius of each power circle, the polycrystalline model is converted to Voronoi tessellation.

**Figure 2:**
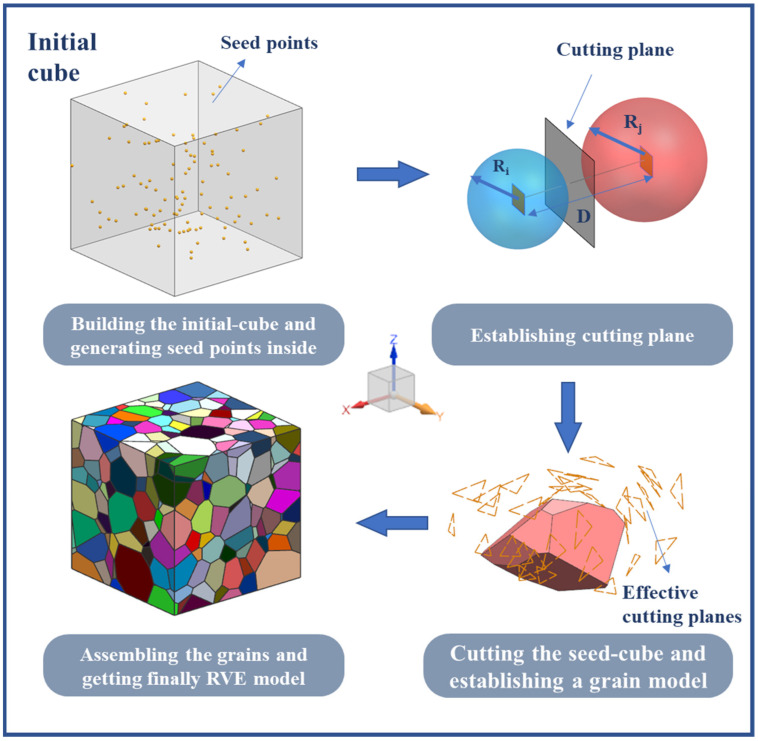
Flow chart of polycrystal modeling.

**Figure 3:**
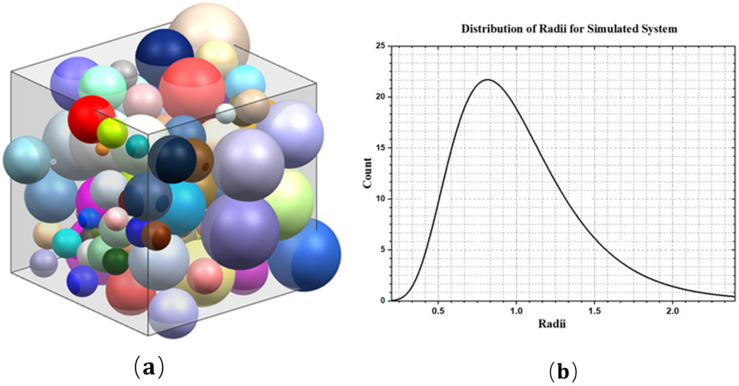
(**a**) Sphere packing of 100 lognormally distributed sphere; (**b**) histogram of radii plotted the lognormal distribution.

**Figure 4:**
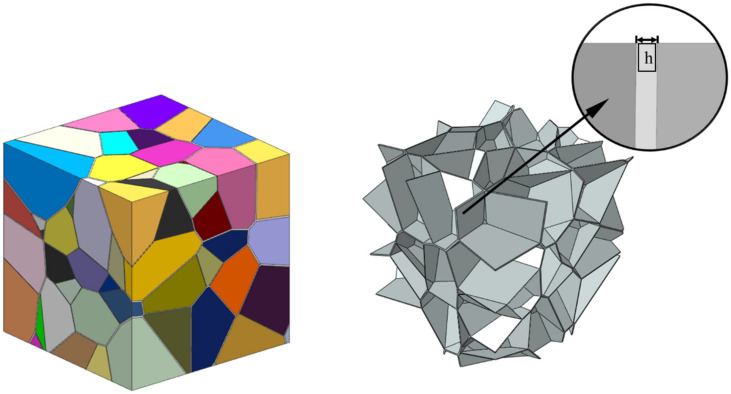
The polycrystalline microstructure model with grain boundary thickness h.

In this paper, a new polycrystalline microstructure modeling method was implemented. The flow chart is shown in Figure 2. Firstly, spherical particles corresponding to the number of grains are randomly generated in the RVE region, and the positions and radius sizes of these particles are adjusted so that they meet the stacking requirements [16,17], as shown in Figure 3. The seed points are established using the spherical center coordinates of the spherical particles. Secondly, a 3D Delaunay triangulation is created from the seed points. According to the data structure of vertices and edges in the Delaunay triangulation network, the adjacent seed points around each seed point which will achieve an effective cut on its cells can be efficiently determined. Thirdly, the corresponding cube is built by taking a seed point as the center and establishing the cutting planes between this seed point and its adjacent seed points. The grain model is obtained by cutting the corresponding cube with all the established cutting planes. Finally, Loop the above steps to obtain the grain model of each seed point, and a Boolean operation is conducted between each grain with the original cube to obtain the 3D grain boundary model; the program terminates when the final polycrystalline model is obtained (Figure 4).

The procedure can be presented as follows:

The input and output of algorithm are as follows:

Input: The coordinates of the seed points $\left\{\left(x_{i},y_{i},z_{i}\right)\left|i=1,2,3\ldots \right.\right\}$, radius sizes of spheres $\left\{r_{1},r_{2},r_{3}\ldots \right\}$ and thickness of the grain boundary h.

Output: Achieving the establishment of polycrystalline material models with solid grain boundary model.

1. Create initial cube and seed points
2. Delaunay triangulation of seed points and determine the effective cutting seed points around each seed point
3. While the number of loops less than of seed points:
  3.1. Select a seed point to create a seed point cube
  3.2. Create cutting planes
    If the cutting plane intersects with the grain model:
      Cutting the seed cube with cutting plane
    Else:
      Finding next cutting plane
    If there is no cutting plane
    End of if
    Go back to (3)
  End While
4. Boolean operation between each grain and the original cube to get the thickness of *h* grain boundary model
5. Return Final Model

**Figure 5:**
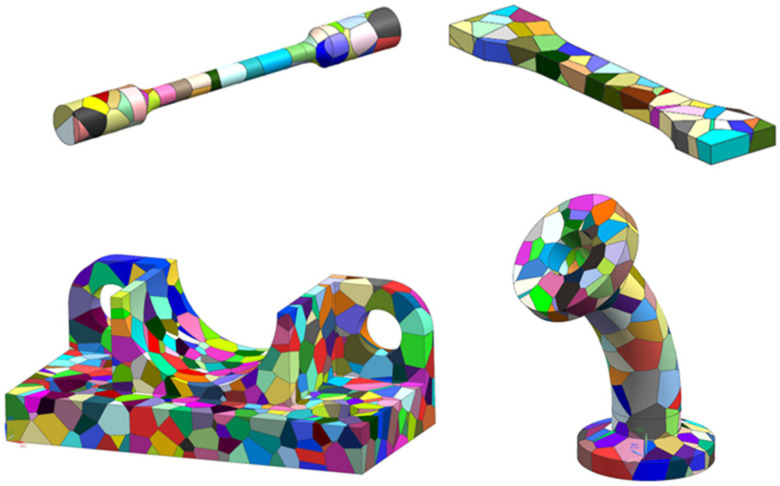
Examples of arbitrary solid shapes with Voronoi cells.

In particular, because of the presented formulation being implemented in the CAD package, it is possible to tessellate arbitrary desired solid models with Voronoi cells where the seed points are assigned randomly, as shown in Figure 5.

## 3. Finite Element Modeling of Polycrystalline Microstructures

In this section, the finite elements model of the polycrystalline microstructure is created in the commercial software ABAQUS (6.14, 2017, Dassault Systemes, Paris, France), including material properties, grain assembly process, boundary conditions, and meshing. Based on this finite element model, the macroscopic elastic properties of polycrystalline ceramic materials will be simulated in Section 4.

### 3.1. Material Model

In this paper, each grain in the polycrystalline model was considered to be a three dimensions linear elastic anisotropy domain with arbitrary orientation. For general elastic anisotropic materials, the elastic constants relate the stress σ to the strain ε through the generalized Hooke’s law. Using the compact Voigt notation, the stress–strain relation can be expressed as follows:

(6) $$\sigma_{mn}=C_{mnkl}\varepsilon_{kl}\Rightarrow \sigma_{i}=C_{ij}\varepsilon_{j}$$

or

(7) $$\varepsilon_{i}=S_{ij}\sigma_{j}$$

The mapping of the tensor indices mn or kl to the matrix indices i or j is

(8) $$\begin{array}{l} 11\Rightarrow 1\\ 22\Rightarrow 2\\ 33\Rightarrow 3\\ 23=32\Rightarrow 4\\ 13=31\Rightarrow 5\\ 12=21\Rightarrow 6 \end{array}$$

where $C_{ij}$ is the stiffness tensor, $S_{ij}$ is the flexibility tensor, and the grain model is a three-dimensional finite element model, so the stiffness matrix is

(9) $$\{\begin{array}{c} \sigma_{1}\\ \sigma_{2}\\ \sigma_{3}\\ \sigma_{4}\\ \sigma_{5}\\ \sigma_{6} \end{array}\}=[\begin{array}{cccccc} C_{11} & C_{12} & C_{13} & C_{14} & 0 & 0\\ C_{12} & C_{11} & C_{13} & -C_{14} & 0 & 0\\ C_{13} & C_{13} & C_{33} & C_{11} & 0 & 0\\ C_{14} & -C_{14} & 0 & C_{44} & 0 & 0\\ 0 & 0 & 0 & 0 & C_{44} & C_{14}\\ 0 & 0 & 0 & 0 & C_{14} & \frac{1}{2}(C_{11}-C_{12}) \end{array}]\{\begin{array}{c} \varepsilon_{1}\\ \varepsilon_{2}\\ \varepsilon_{3}\\ \varepsilon_{4}\\ \varepsilon_{5}\\ \varepsilon_{6} \end{array}\}$$

where the stress σ and strain ε are represented in vector form as 6 × 1 matrices. The $C_{14}$ is only related to complex deformations, but there are only simple tensile and shear deformation calculations considered in this paper, and the value of $C_{14}$ is small compared to other stiffness values, so it is approximated as zero [18]. The flexibility matrix is $S_{ij}=C_{ij}{}^{-1}$. Finally, a local coordinate system is independently assigned to each grain by a Python Scripts for ABAQUS, and a randomly distributed grain orientation is generated by the random factor method and assigned to each grain separately.

In the current study, ${\mathrm{Al}}_{2}O_{3}$ ceramic materials were investigated as elastic materials because their plasticity has a negligible effect on the behavior of the material. Moreover, there is a large number of consistent measurements of single crystal properties in the literature, and the limited anisotropy of single crystals. In this paper, we determined $C_{11}=497.6\;\mathrm{GPa},\;C_{12}=162.6\;\mathrm{GPa},\;\;C_{13}=117.2\;\mathrm{GPa},\;C_{33}=498.1\;\mathrm{GPa},\;C_{44}=C_{55}=147.2\;\mathrm{GPa}$, according to reference [18].

### 3.2. Assembling the Grains

**Figure 6:**
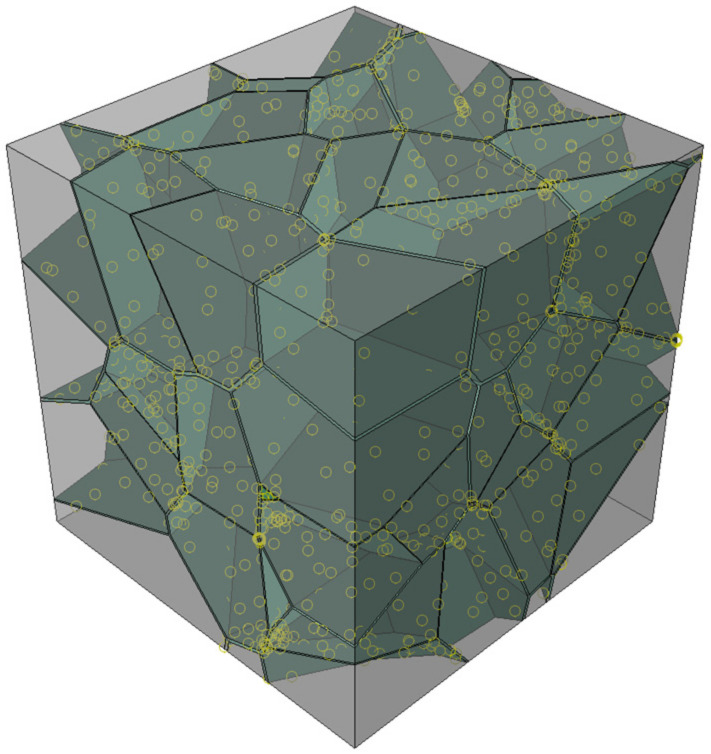
Assembling parts through cohesive contacts.

The single grains in polycrystalline models established by this presented method are independent entities, so it is essential to assemble them together with cohesive contacts to simulate the viscous connection between the grains. In this paper, the grains and grain boundaries were assembled together by setting the viscous property through cohesive contacts between each pair of contact surfaces through subroutine programming. The method in this paper was as follows: First, use the Find Contact Pairs module to create a surface-to-surface cohesive surface between each contact pair, then determine whether the Master surface and Slave surface of each contact pair are consistent, and then delete the inconsistent contact pairs, and finally obtain the results as shown in [19] Figure 6.

### 3.3. Boundary Conditions

During the numerical homogenization calculations for polycrystalline ceramic materials, the boundary conditions imposed on the RVE must satisfy the energy conservation criterion for the micro–macro scale transition proposed by Hill’s energy law [16].

(10) $$\langle \sigma :\varepsilon \rangle =\langle \sigma \rangle :\langle \varepsilon \rangle$$

In this paper, six different groups of boundary conditions were chosen to impose RVE in the simulation of homogenization of ceramic materials under small elastic deformations.

(11) $$\begin{array}{l} \varepsilon_{11}=\bar{\varepsilon },\varepsilon_{22}=0,\varepsilon_{33}=0,\varepsilon_{12}=0,\varepsilon_{13}=0,\varepsilon_{23}=0\\ \varepsilon_{11}=0,\varepsilon_{22}=\bar{\varepsilon },\varepsilon_{33}=0,\varepsilon_{12}=0,\varepsilon_{13}=0,\varepsilon_{23}=0\\ \varepsilon_{11}=0,\varepsilon_{22}=0,\varepsilon_{33}=\bar{\varepsilon },\varepsilon_{12}=0,\varepsilon_{13}=0,\varepsilon_{23}=0\\ \varepsilon_{11}=0,\varepsilon_{22}=0,\varepsilon_{33}=0,\varepsilon_{12}=\frac{\bar{\varepsilon }}{2},\varepsilon_{13}=0,\varepsilon_{23}=0\\ \varepsilon_{11}=0,\varepsilon_{22}=0,\varepsilon_{33}=0,\varepsilon_{12}=0,\varepsilon_{13}=\frac{\bar{\varepsilon }}{2},\varepsilon_{23}=0\\ \varepsilon_{11}=0,\varepsilon_{22}=0,\varepsilon_{33}=0,\varepsilon_{12}=0,\varepsilon_{13}=0,\varepsilon_{23}=\frac{\bar{\varepsilon }}{2} \end{array}$$

**Figure 7:**
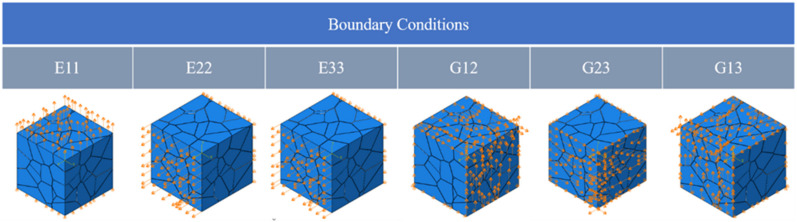
Boundary conditions for the RVE model, E11, E22, E33 represent the tensile boundary conditions in X, Y and Z directions respectively, G12, G23, G13 are the shear boundary conditions in XY, YZ and XZ directions respectively.

The achieved result is shown in Figure 7.

### 3.4. Mesh of the Grains

**Figure 8:**
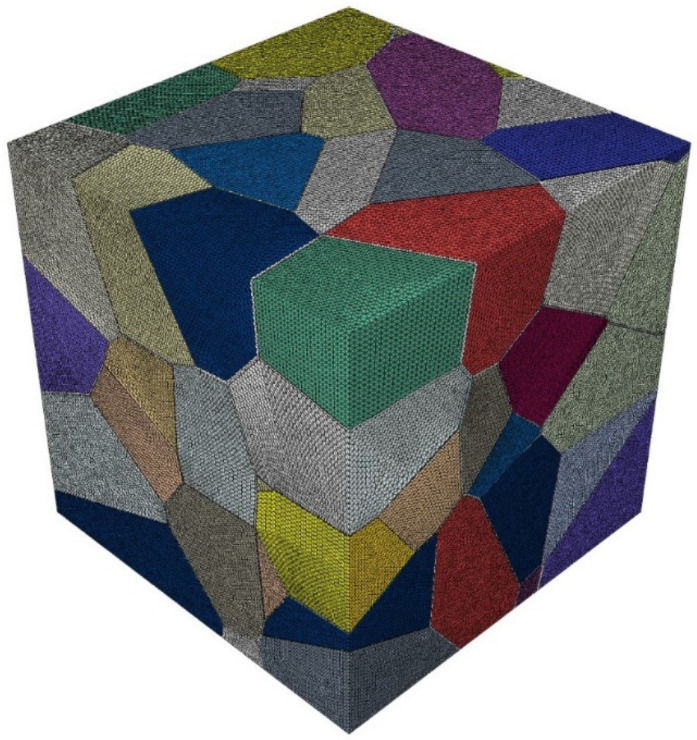
Fully assembled FE model of a 100-grain specimen.

The mesh discretization method in this paper was based on a discretization approach based on a widely used bottom-up procedure consisting of mesh nodes, edges, faces, and particles (i.e., 0D, 1D, 2D, and 3D entities) in that order. The 0D meshing consists of specifying a feature length for each node. Each edge is then discretized in a 1D element, with the length derived from the feature length of both vertices. A feature length that determines the length of the 2D elements is specified for each new node, and then each face is meshed into a triangular element. The same procedure is applied to the 3D meshing; each particle is discretized into tetrahedral cells [13]. Depending on the quality of the mesh, the tetrahedral cell can be either C3D4 (4 nodes) or a secondary cell C3D8R (10 nodes). The meshing diagram of this paper is shown in Figure 8.

### 3.5. Finite Element Homogenization Scheme

The RVE-based finite element homogenization method is an excellent method to predict the effective mechanical properties of various polycrystalline microstructure materials. In this paper, the finite element homogenization method was used to predict the elastic modulus of ${\mathrm{Al}}_{2}O_{3}$ ceramic materials. The preprocessing (including boundary condition, material properties, etc.), finite element analysis, and postprocessing corresponding to the evaluations on the mechanical properties of the polycrystalline ${\mathrm{Al}}_{2}O_{3}$ ceramic materials are implemented in the finite element package ABAQUS [20]. Finite element homogenization calculations are implemented in the commercial FEM package ABAQUS.

The effective mechanical properties are intrinsic to the materials and independent of the external effects, such as body force and boundary condition. Therefore, to predict the effective mechanical properties of polycrystalline materials by using the RVE-based finite element homogenization method, the following weak form quasi-static equilibrium equation is considered:

(12) div(σ(x))=0

Moreover, the boundary conditions are necessary for solving the quasi-static equilibrium equation (Equation (12)) and must satisfy Hill’s energy law. Here, Hill’s energy law states that the energy on the micro-level has to be the same as the effective energy for the homogenization [21]. For any material point ***x*** in the RVE, its constitutive model is given as

(13) σ(x)=σ(x,ε(x))

The weak form quasi-static equilibrium equation is solved based on the finite element analysis in the RVE, with the boundary condition satisfying Hill’s energy law (Equation (10)) and the constitutive relationship (Equation (13)).

During the postprocessing, the stress tensor, strain tensor, and volume (IVOL) of anyone integration point of RVE can be obtained in finite element analysis, and the average stress $\langle \sigma_{ij}\rangle$ and strain $\langle \varepsilon_{ij}\rangle$ can be calculated by Equation (14):

(14) $$\begin{array}{l} \langle \sigma_{ij}\rangle =\frac{1}{V}\sum_{e=1}^{n_{e}}V_{e}\left[\sum_{I=1}^{n_{e\mathrm{int}}}\sigma_{ij}(y_{I})\cdot J(y_{I})\cdot W(y_{I})\right]\\ =\frac{1}{V}\sum_{I=1}^{n_{\mathrm{int}}}\sigma_{ij}(y_{I})\cdot IVOL\;(y_{I})i,j=1,2,\cdots \;and\;y_{I}\in V\\ \langle \varepsilon_{ij}\rangle =\frac{1}{V}\sum_{e=1}^{n_{e}}V_{e}\left[\sum_{I=1}^{n_{e\mathrm{int}}}\varepsilon_{ij}(y_{I})\cdot J(y_{I})\cdot W(y_{I})\right]\\ =\frac{1}{V}\sum_{I=1}^{n_{\mathrm{int}}}\varepsilon_{ij}(y_{I})\cdot IVOL\;(y_{I})i,j=1,2,\cdots \;and\;y_{I}\in V\; \end{array}$$

where $n_{e}$ is the number of elements; $n_{e\;\mathrm{int}}$ and $n_{\mathrm{int}}$ are the numbers of the integration point in the elements *e* and the integral RVE (*V*), respectively; and $J\left(y_{I}\right)$ and $W\left(y_{I}\right)$ are the Jacobian matrix and weight matrix, respectively, at an integration point located at $y_{I}$ in the element *e*, which occupies a domain size of $V_{e}$. $IVOL\left(y_{I}\right)$ is the volume of the integration point at an integration point positioned at $y_{I}$ [20].

In this paper, the linear elastic deformation was considered for ceramic materials; the elastic stiffness tensor $\langle C\rangle$ can be computed by the average stress $\langle \sigma_{ij}\rangle$ and average strain $\langle \varepsilon_{ij}\rangle$ of the RVE (Equation (15)).

(15) $$\langle C\rangle =\langle \sigma \rangle {\langle \varepsilon \rangle }^{-1}$$

## 4. Results and Discussion

In this section, statistical analysis and various finite element models of different polycrystalline models are used to illustrate the capability of the presented method for producing statistically representative microstructures.

Firstly, statistical analysis of grain face and grain size distribution are performed with the polycrystalline model. Compared with the Voronoi tessellation, the grain face and grain size distributions are obviously lognormal distribution.

Then, the polycrystalline model is assigned with ${\mathrm{Al}}_{2}O_{3}$ linear elastic anisotropy properties, and the effective mechanical parameters are calculated by finite element homogenization method. The numerical results are compared with experimental measurement to validate the representativeness of the polycrystalline models.

### 4.1. Microstructure Analysis

#### 4.1.1. The Distribution of Grain Size

The grain size distribution in real polycrystalline materials has been suggested to be lognormal in the previous studies [22,23,24]. Its probability density function is described as

(16) $$f(x;\mu ,\sigma )=\frac{1}{x\sigma \sqrt{2\pi }}e^{\frac{-{\left(\ln x-\mu \right)}^{2}}{2\sigma^{2}}},x>0$$

where σ and μ are standard deviation and arithmetic mean of ln***x***, respectively.

For the purposes of comparison, two sets of polycrystalline microstructure models are generated separately in RVE based on the Voronoi and Laguerre–Voronoi diagram. To explore the effect of the number of grains, each group consists 125 and 500 grains. In order to have statistically valuable number results, ten different models—for a given number of grains (e.g., 125 and 500 grains)—were generated.

**Figure 9:**
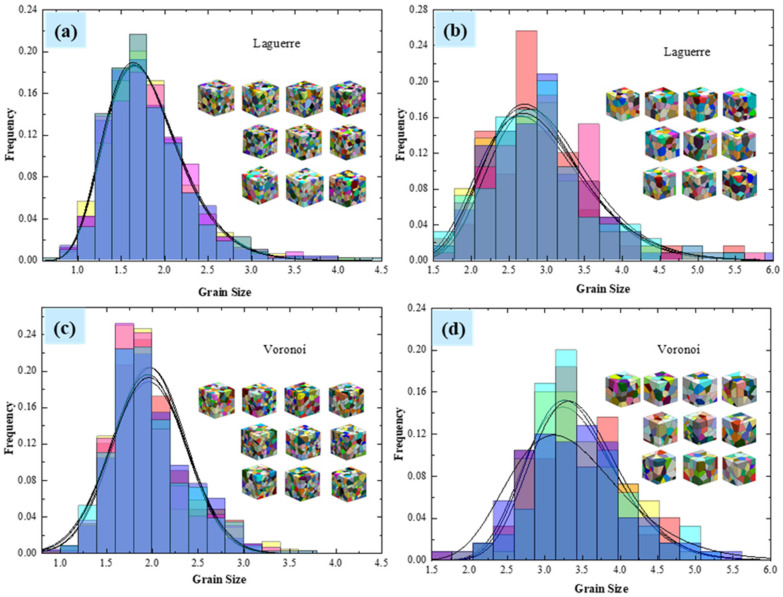
(**a**) Grain size distribution of the Laguerre–Voronoi tessellation polycrystalline model with 500 grains; (**b**) grain size distribution of the Laguerre–Voronoi tessellation polycrystalline model with 125 grains; (**c**) grain size distribution of the Voronoi tessellation polycrystalline model with 500 grains; (**d**) grain size distribution of the Voronoi tessellation polycrystalline model with 125 grains.

During the statistical analysis, the grain size distributions (Figure 9) show that the grain size of the Voronoi tessellation-based polycrystalline model has a Gaussian normal distribution,

(17) $$y=y_{0}\;+\;\left(A/\left(\sigma \cdot \mathrm{sqrt}\left(2\pi \right)\right)\right)\cdot \exp\left(-2\cdot {\left(\left(x-\mu \right)/2\sigma \right)}^{2}\right),$$

and the Laguerre–Voronoi tessellation-based one has a log-normal distribution:

(18) $$y\;=\;y_{0}+A/\left(\mathrm{sqrt}\left(2\pi \right)\sigma x\right)\cdot \exp\left(-{\left(\ln x-\mu \right)}^{2}/\left(2\sigma^{2}\right)\right),$$

where the σ and μ are standard deviation and arithmetic mean, respectively.

With the increasing of the number of grains, the grain size distributions of the polycrystalline models seem to become more stable, and the fitting degree is higher. Compared with the Voronoi tessellation-based polycrystalline models, the Laguerre–Voronoi tessellation-based ones allowing a wider range of grain structures to be modelled.

#### 4.1.2. The Distribution of Face and Edge Number

**Figure 10:**
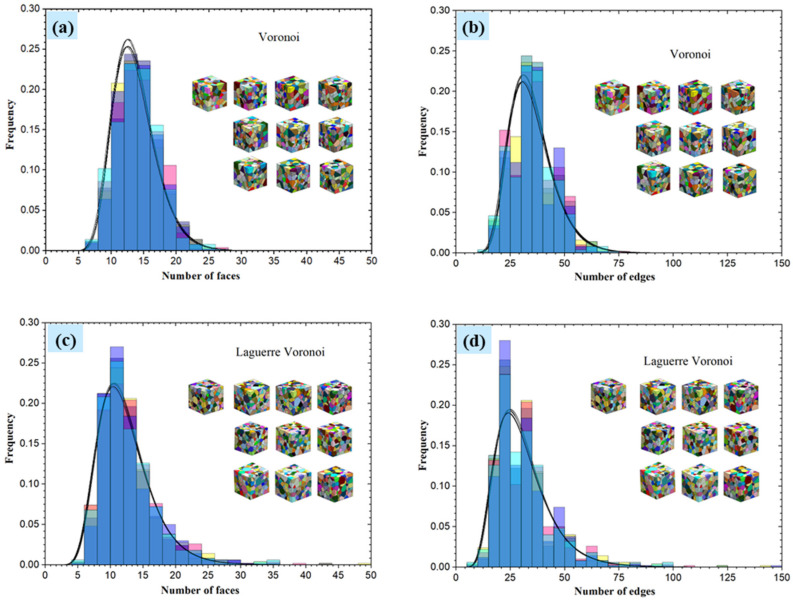
(**a**) Distribution of the number of grains faces, (**b**) distribution of the number of single grain edges by Voronoi tessellation, (**c**) distribution of the number of single grain faces, (**d**) distribution of the number of single grain edges by Laguerre–Voronoi tessellation.

Statistical analysis for the number of grain faces and edges was also performed for a polycrystalline model with 500 grains, and the statistical results are shown in Figure 10. The number of grains faces in polycrystalline models is concentrated between 10 and 15, and the number of edges concentrates between 25 and 40, which is consistent with the actual grain microscopic morphology [2,5]. Furthermore, as with the distribution of grain size, the distribution functions of the number of grain faces and edges are fitted to the normal and log-normal distribution functions, respectively.

**Figure 11:**
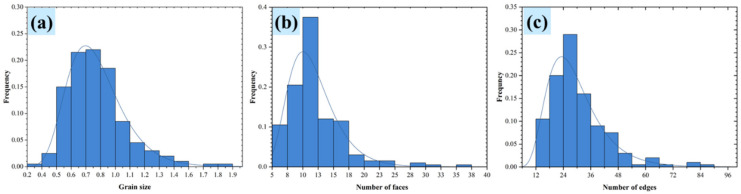
(**a**) The distribution of grain sizes, (**b**) the distribution of the face number, and (**c**) the distribution of the edge number of the 200-grain polycrystal model.

In addition, the 3D grain boundary can also be constructed in the polycrystalline model by the presented method in this paper. The distributions of grain size, and the number of grains faces and edges is shown in Figure 11 for a grain boundary thickness *h* of 0.1 and a number of grains of 200. The results show that the distributions of grain size, and the number of grain faces and edges of polycrystalline model grains that have solid grain boundaries and established by the Laguerre–Voronoi method conform to the log-normal distribution function shown in Equation (18). Moreover, the grain topology information in Figure 11 is consistent with the experimentally measured grain information in a statistically regular manner [25].

### 4.2. Predicting the Effective Elastic Modulus of Al_2_O_3_ Ceramic Materials

For predicting the elastic modulus of ${\mathrm{Al}}_{2}O_{3}$ ceramic materials, linear elastic deformation is considered, there no material plasticity, and there are no defects. The effects of grain size, grain orientation, and grain boundary in the polycrystalline model to predict the effective elastic modulus of ${\mathrm{Al}}_{2}O_{3}$ ceramic materials are investigated in this section.

#### 4.2.1. Effect of Grain Size

An RVE of a polycrystalline model usually requires enough number of grains to convey consistent bulk properties. In order to investigate the adequate number of grains to construct an RVE of polycrystalline ceramic materials, different polycrystalline microstructure models with different numbers of grains are generated. Benedetti [20] found that polycrystalline finite anisotropic materials (e.g., nickel, copper, gold) with cubic grain structures can be calculated with 20 grains for elastic properties within 10% error under specific boundary conditions. The ${\mathrm{Al}}_{2}O_{3}$ ceramic materials have finite anisotropy with linear elastic properties, so the minimum RVE structure is defined as consisting of 25 grains in this paper.

**Figure 12:**
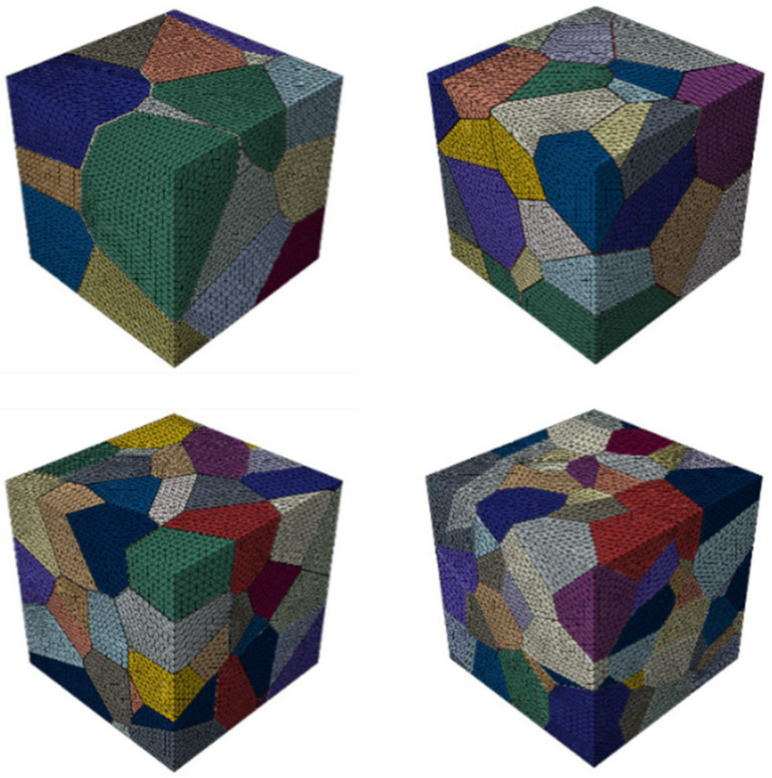
Finite element model of polycrystals with grain numbers of 25, 50, 100, and 200.

In this section, finite element models with 25, 50, 100, 200, and 500 grains are established to predict the effective elastic modulus of ${\mathrm{Al}}_{2}O_{3}$ ceramic materials (Figure 12), and 10 different topologies are established for the given number of grains to reduce the influence of random errors on the calculation results.

**Figure 13:**
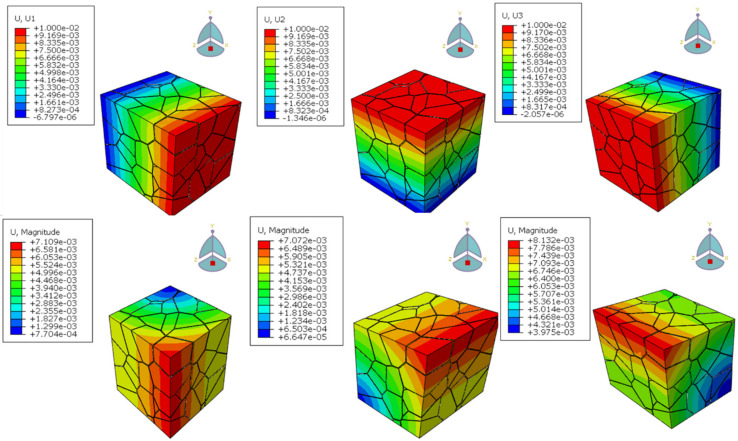
Deformation of 100-grain ${\mathrm{Al}}_{2}O_{3}$ ceramic material under the boundary conditions.

According to references [26,27,28], the elastic modulus interval of ${\mathrm{Al}}_{2}O_{3}$ material is obtained, as shown in Table 1. The polycrystalline model in this paper is an ideal model and does not consider defects such as pores and microcracks. Therefore, the maximum value of 410 in the interval is selected as a reference, that is, E=410 GPa, to evaluate the accuracy. Table 2 shows the effective elastic modulus of ${\mathrm{Al}}_{2}O_{3}$ ceramic materials predicted with these four sets of RVE models. $E_{11}$, $E_{22}$, and $E_{33}$ are the average values of elastic modulus in *x*, *y*, and *z* directions for 10 different topologies, respectively, and the relative error $e_{ii}$ is calculated using Equation (19). It can be concluded that the effective elastic moduli are all distributed within the same ranges of values measured experimentally and reported in the literature [21]. With the increase of the number of grains, the errors of calculation results gradually decrease. Considering the balance between computational cost and accuracy, for a grain number larger than 100, the effect of single grain spatial distribution on the stress–strain response or overall elastic modulus can be ignored. The deformation of the RVE model of polycrystalline material with 100 grains under the six kinds of boundary conditions in this paper is shown in Figure 13.

(19) $$e_{ii}=\frac{E_{ii}-E}{E}$$

#### 4.2.2. Effect of Grain Orientation

The grain orientation is determined by the order of atomic arrangement, and the atoms are randomly arranged during grain growth, so the polycrystalline materials are usually intergranular heterogeneous in microstructure, and the grain orientation can be simulated by the random factor method. However, the bulk properties of polycrystalline materials are isotropic. According to Section 4.2.1, the adequate number of grains is taken as 100, and the effect of grain orientation on the effective elastic modulus is investigated in this section by assigning different grain orientations to the same finite element models.

**Figure 14:**
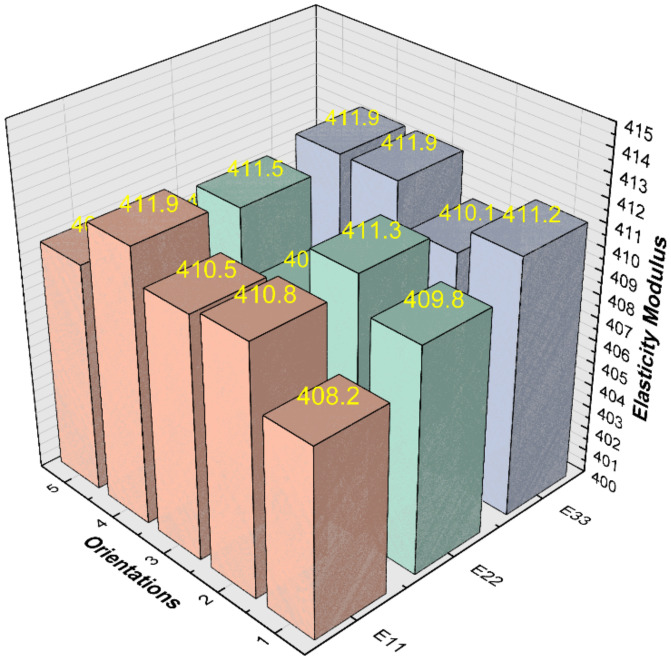
Effective elastic modulus predicted for five different grain orientations.

In this section, the grain orientations are generated by the random factor method with randomly distributed orientations for each grain, and five different sets of grain orientations are assigned to the identical polycrystalline finite element models. Then, the effective elastic modulus of each model in three different directions is obtained, and the calculation results are shown in Figure 14.

When the number of grains is 100, the effective moduli of elasticity for different grain orientations are in the range of 407–412 GPa and the average modulus of elasticity is within 1% error from the value obtained according to reference [26,27,28] (E=410 GPa). It can be concluded that the grain orientation constructed by the random factor method can be used to simulate the randomness of grain orientation in the polycrystalline materials.

#### 4.2.3. Effect of Grain Boundary

The difference in grain orientation leads to the formation of grain boundaries between adjacent grains during the grain growth process, and the existence of grain boundaries is the fundamental reason for the discontinuity of material properties, so it is often regarded as a weak point of material structure in the study of material structure. Nowadays, due to the small size of grain boundaries and the complex chemical composition, structure, and mechanical environment, there is no direct and accurate quantitative analysis method and experimental data for the thickness of grain boundaries and various material parameters. Therefore, the material properties of 3D grain boundaries are simplified as isotropic in this paper, and the elastic modulus of 3D grain boundaries are considered in three cases:

1. The elastic modulus of the grain boundaries is 50% of the single crystal of the ${\mathrm{Al}}_{2}O_{3}$ ceramic material.
2. The elastic modulus of the grain boundaries is consistent with the elastic modulus of the ${\mathrm{Al}}_{2}O_{3}$ ceramic material.
3. The elastic modulus of the grain boundaries is 150% of the single crystal of the ${\mathrm{Al}}_{2}O_{3}$ ceramic material.

**Figure 15:**
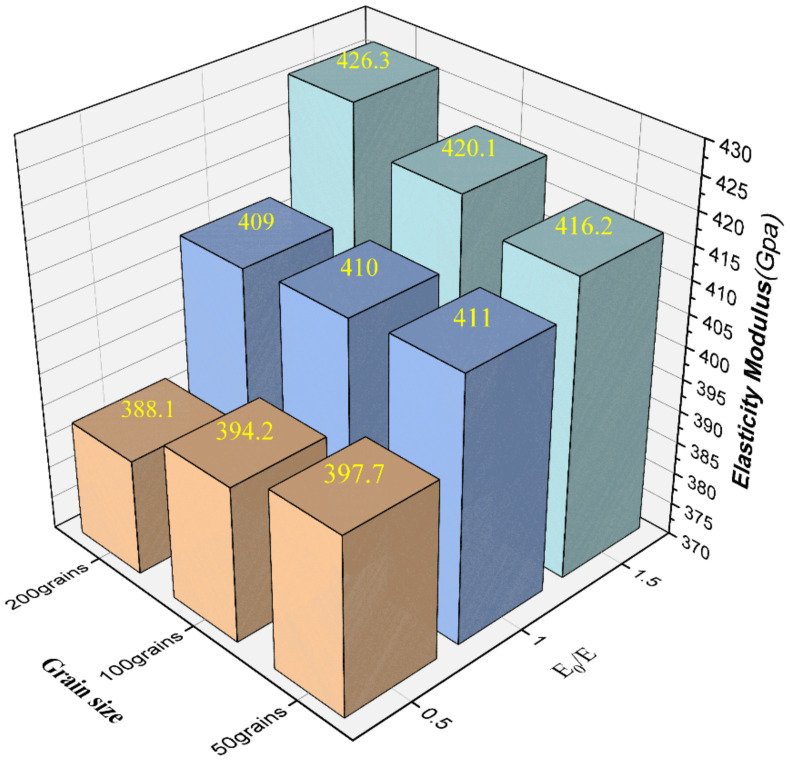
Effective elastic modulus at different grain sizes for three different grain boundary elastic moduli.

For the three cases, the effective elastic modulus at different grain sizes was given as shown in Figure 15. In the figure, $E_{0}$ is the elastic modulus of grain boundaries, and E is the single crystal elastic modulus of ${\mathrm{Al}}_{2}O_{3}$. When the value of $E_{0}/E$ is 1.5 where the grain boundaries are considered to hinder grain deformation in the actual polycrystalline material, the overall macroscopic elastic modulus is strengthened as the average grain size decreases (the number of grains in the RVE model increases) where the density of grain boundaries increases, which is in accordance with the Hall–Petch equation. When the value of $E_{0}/E$ is 0.5, with the decrease of grain size, the volume fraction of grain boundaries increases, and the macroscopic elastic modulus becomes smaller with grain refinement to achieve the toughening effect. It is concluded that when predicting the mechanical properties of polycrystalline materials, the presence of solid grain boundaries in the polycrystalline model results in the calculations being more consistent with the mechanical properties of the actual materials.

## 5. Conclusions

A new implementation of the Voronoi diagram in Laguerre geometry is presented in this paper for the generation of numerical models of polycrystalline microstructures, where the size and shape of the grains can be controlled, and the 3D grain boundaries can be modeled with a specified thickness. This method directly models the grains in CAD software. There is no need for cumbersome data processing and reverse topology reconstruction. Moreover, the resulting models can be directly imported into various finite element packages (e.g., ABAQUS, ANSYS, COMSOL, etc.) for the simulation and analysis of polycrystalline materials.

In order to verify the capability of the presented method, the statistical analysis of grain size, grain face, and grain edge distribution are performed with the established polycrystalline models, and the macroscopic elastic properties of polycrystalline ceramic materials are simulated. It is shown that the grain size, grain face, and grain edge distribution can be fitted to a lognormal distribution, compared with the normal distribution in Voronoi-based tessellation methods.

The effective elastic modulus of polycrystalline ceramic materials is predicted by the RVE-based finite element homogenization method. The effects of grain size, grain orientation, and grain boundary in the polycrystalline model materials are investigated. The predicted effective elastic moduli are compared with the experimental measurement, and the validity of the proposed method is verified.

## Figures and Tables

**Table 1:** Material properties of ${\mathrm{Al}}_{2}O_{3}.$

Material	*μ* (GPa)	*B* (GPa)	*v*	*E* (GPa)
Al_2_O_3_(*w* > 99.9%)	163	251	0.233	366–410

**Table 2:** Elasticity modulus and relative error of RVE models.

Grain Number	Elastic Modulus (GPa)			Relative Error			Computation Time (sec)
	*E*_11_	*E*_22_	*E*_33_	*e*_11_	*e*_22_	*e*_33_	*t*
25	415.3	405.6	401.3	1.29%	1.07%	2.12%	89.7
50	405.9	411.4	408.6	1.00%	0.34%	0.34%	234.0
100	410.0	407.0	407.4	0.00%	0.73%	0.63%	553.5
200	408.3	407.7	408.2	0.41%	0.56%	0.43%	1789.5
500	409.7	409.3	409.6	0.073%	0.17%	0.097%	3457.9

## Data Availability

The data that support the findings of this study are available from the corresponding author upon reasonable request.
